# Nurse stress relief: A pilot study on the impact of healing touch and chakra techniques on well-being

**DOI:** 10.1097/NSG.0000000000000117

**Published:** 2024-12-20

**Authors:** Stacy A. Noel, Danielle Altares Sarik, Michelle Collins

**Affiliations:** At ChristianaCare in Wilmington, Del., **Stacy Noel** is a nursing integrative care manager, **Danielle Sarik** is a nurse scientist, and **Michelle Collins** is the vice president for nursing excellence.

**Keywords:** burnout, chakra, healing touch, nurses, self-care, stress management

## Abstract

**Purpose:**

To investigate and assess the effects of Healing Touch (HT) level-one education and training and the 21-day practice of the self-chakra connection technique on the perceived stress levels of nurses at a large healthcare system located in the Mid-Atlantic US region.

**Methods:**

This study used a mixed-methods design to collect quantitative and qualitative data via pre- and post-intervention surveys with Cohen's Perceived Stress Scale and post-open-ended questions. Data were analyzed using descriptive and correlational statistics.

**Results:**

In this pilot study of 58 nurses who participated in HT classes, 34 nurses completed the presurvey and 22 completed both pre- and post-surveys. Results revealed a 3.8-point mean decrease in perceived stress scores, with participants reporting improved stress management, increased confidence, and better work preparedness. However, statistical significance was not achieved due to low post-survey completion rates.

**Conclusion:**

Nurses who received HT training and practiced the self-chakra connection technique as part of their self-care reported decreased perceived stress levels, supporting the use of HT as a potentially effective approach to stress reduction among nurses.

Nursing can be a physically, emotionally, and mentally demanding profession that places nurses at risk for burnout,^1^ which has profound effects on patients, caregivers, and health systems. Nurse turnover related to stress and burnout is costly, with the average replacement costs ranging from $40,000 to $100,000 per nurse.^2^

High work-related stress and burnout contribute to nurses leaving the profession, exacerbating existing nursing shortages.^1^ Factors reported by nurses that contribute to high stress and burnout are the work environment and multiple demands.^1^ Promoting stress management strategies such as resiliency, stress management, and mindfulness-based training has been shown to improve nurses' health, job satisfaction, work performance, retention, and patient satisfaction.^3^-^6^

Many nursing and medical organizations promote stress reduction and self-care. The American Nurses Association Scope and Standards of Practice, 4th edition^7^ stresses the importance of caring for one's well-being as a priority of all nurses. The National Academy of Medicine^8^ provides evidence-based key steps to improve clinician well-being, enhance performance, and promote patient safety.

One innovative approach to address nurse stress and burnout is the practice of Healing Touch (HT), an energy-based therapy that teaches gentle touch techniques to promote relaxation and support stress reduction.^9^,^10^

HT is founded on the belief that HT techniques assist in restoring harmony and balance to an individual's energy system, thereby placing the individual in a position to support their ability to heal.^10^-^13^ HT is believed to impact the mind-body connection, which may promote relaxation and stress reduction.^10^-^13^

Research on the use of HT has shown some promising benefits, including reductions in pain, anxiety, and stress, as well as promotion of relaxation and overall health. However, though preliminary findings are encouraging, there remains a need for more rigorous, high-quality, peer-reviewed research to confirm the efficacy and long-term benefits of HT in clinical settings.^9^-^12^

The Healing Touch Program (HTP) Code of Ethics focuses on self-care as a part of the practitioner's self-development.^14^ In HT, practitioners practice self-care to enhance their health and to be able to provide optimal care to others.^13^,^14^ HT techniques can also be used on friends, family members, patients, and as part of self-care.^13^,^14^

HT classes present information about the human energy systems within and around the body.^13^-^16^ These energy systems include chakras, biofield or aura, and meridians.^13^-^16^ Chakras are energy centers that emanate from the spine through the body.^13^-^16^ There are seven main charkas, each one with a corresponding number, name, color, specific location in the body, organs, endocrine glands, nerves, and health focus.^13^-^16^ Minor energy chakras are located in each joint.^13^-^15^

The biofield or aura is theorized as the electromagnetic field emitted from the body.^9^,^15^,^16^ The biofield has seven layers, with each layer correlating to a different element of the human being's physical, mental, spiritual, and emotional aspects.^13^-^16^

Meridians are energy pathways that run through the body and connect to different organ systems.^13^-^16^ Acupuncture and acupressure focus on improving the flow of energy in the meridians, potentially alleviating various clinical conditions.^13^-^16^

HT classes teach participants how to assess the chakras, biofield/aura, and meridians by completing a hand scan and using a pendulum.^14^

This article discusses a mixed-methods study on the impact of HT training and practice of the self-chakra connection (SCC) technique as a self-care method on nurses' perceived stress at a large Mid-Atlantic healthcare system with multiple Magnet^®^-recognized entities. The institution offered HT classes every week. The SCC is a full-body technique believed to facilitate the balancing of the human energy system to promote relaxation.

**Table:** Perceived Stress Scale^22^

The questions in this scale ask you about your feelings and thoughts during THE LAST MONTH. In each case, please indicate your response by circling the number representing HOW OFTEN you felt or thought a certain way.						
**0 = Never 1 = Almost Never 2 = Sometimes 3 = Fairly Often 4 = Very Often**						
1.	In the last month, how often have you been upset because of something that happened unexpectedly?	0	1	2	3	4
2.	In the last month, how often have you felt that you were unable to control the important things in your life?	0	1	2	3	4
3.	In the last month, how often have you felt nervous and “stressed”?	0	1	2	3	4
4.	In the last month, how often have you felt confident about your ability to handle your personal problems?	0	1	2	3	4
5.	In the last month, how often have you felt that things were going your way?	0	1	2	3	4
6.	In the last month, how often have you found that you could not cope with all the things that you had to do?	0	1	2	3	4
7.	In the last month, how often have you been able to control irritations in your life?	0	1	2	3	4
8.	In the last month, how often have you felt that you were on top of things?	0	1	2	3	4
9.	In the last month, how often have you been angered because of things that were outside of your control?	0	1	2	3	4
10.	In the last month, how often have you felt difficulties were piling up so high that you could not overcome them?	0	1	2	3	4

Reprinted with permission from the American Sociological Association

## HT in nursing

HTP was created by Janet Mentgen, a Navy nurse, in the 1980s.^13^,^14^ As of this writing, HTP is a multilevel international education program taught in 32 countries and used in over 100 hospitals.^13^,^14^ The HTP curriculum and techniques are based on the healing arts and holistic nursing principles and consists of five sequential levels that build knowledge and skill.^9^,^13^

HT is a continuing-education program recognized by the American Nurses Credentialing Center^®^, the American Holistic Nurses Association, the Canadian Holistic Nurses Association, and the Watson Caring Science Institute, which promotes the importance of self-care for nurses.^9^,^13^,^14^ Thousands of HT students and certified practitioners provide sessions in private practices, wellness centers, schools, and in their homes for family and friends.^13^,^14^ HT is used in healthcare by nurses, physicians, physical therapists, respiratory therapists, chaplains, social workers, counselors, and massage therapists. The majority of HT students are nurses. The HT level 1 classes cover 12 energy techniques that can be used for self-care and for others.

HT techniques are easy to learn and practice on oneself and others in any environment. HT has no contraindications for use.^9^,^13^,^14^ Research suggests that HT is effective in promoting well-being; decreasing pain, anxiety, stress, cortisol levels, and length of hospital stay; and treating posttraumatic stress disorder in veterans.^11^,^17^,^18^

## Literature review

Despite the lack of robust evidence, hospitals are investing in system-level interventions to reduce stress for nurses.^8^ Nurses contribute to the quality of patient outcomes, and therefore, compromised nurse well-being can contribute to decreased quality of care, safety, and productivity.^19^,^20^ Nurse well-being is aligned with improved patient outcomes.^21^,^22^

HT incorporates Watson's theory of human caring into the theoretical framework, supporting the importance of nurses developing individualized self-care strategies to decrease stress.^13^,^14^,^23^ The Caring Theory sees the individual as a whole and integrates care for the mind, body, and spirit to promote well-being.^19^

Nurses' practice of self-care is considered to regenerate their life energies and increase their capacity to care for others.^19^,^23^ Watson and Mentgen believed that nurses need to care for their physical and spiritual being first to provide care to others.^13^,^14^,^23^ Watson's transpersonal-caring-healing model is based on intentionality that enriches the nurse-patient relationship, which deepens the caring and healing process for everyone.^19^ Intentionality is being present to provide authentic care to an individual and oneself by being genuine, using empathy and self-reflection.^19^ Being fully present and intentional in focusing one's attention when caring for the individual is hypothesized to create an energy field around the individual, increasing the healing process.^13^,^14^

In one study that compared HT with deep breathing among acute care nurses, nurse participants said HT was more effective for reducing stress.^24^ The participants said that HT helped them to relax and feel calm and provided a method of soothing and stress reduction. A literature review by Zhang et al.^25^ identified several holistic practices used to reduce stress: mindfulness, self-care workshops, yoga, massage, and meditation. Initiatives aimed at improving the health and self-care of nurses may also lead to improved perceptions of professional well-being.^13^

Tang et al.^17^ demonstrated improving the well-being of nurse leaders through HT training, indicated by a significant improvement in self-reported stress, depression, anxiety, well-being, sleep, and heart rate variability and coherence.

Studies examining the impact of HT and biofield energy approaches on patients offer similar support for these practices. Conway et al.^11^ demonstrated decreased pain and anxiety in patients who received HT in an inpatient rehabilitation unit. In this study, HT was specifically noted to help patients physically, emotionally, and mentally during their healing process.^11^

Studies have examined the use of HT to relieve pain and anxiety, promote relaxation, decrease depression, accelerate wound healing, and promote well-being in patients.^9^,^11^-^13^ However, the authors have found limited studies that examined the impact of HT on nurse outcomes.

This mixed-methods study aimed to explore and evaluate the impact of HT level-one education, training, and practice of the SCC technique on the perceived stress of nurses.

## Methods

### Study design

The study used a mixed-methods design to collect quantitative and qualitative pre- and post-intervention data.

Quantitative data were collected using a Likert survey and qualitative data were obtained through post-open-ended questions. The sample was drawn from a Mid-Atlantic healthcare system with multiple Magnet entities.

### Sample

A total of 58 nurses within the healthcare system who registered for the HT classes between October 2022 and January 2023 were invited to participate in the study. These nurses employed full-time, part-time, or per diem with the health system met the inclusion criteria.

### Recruitment

The HT class was advertised by email, fliers posted in public areas and nurse break rooms, and a hospital-wide electronic newsletter sent to nurses. Nurses who enrolled were recruited for study participation.

### Intervention

HT classes were held in person over two 8-hour days (16 hours total) of in-person training. Fourteen 2-day classes took place from October 2022 to January 2023. The HT classes taught participants how to assess the chakras, biofield/aura, and meridians; described the history of the HT program and practice; introduced participants to Janet Mentgen; and taught 12 energy techniques.

During the 2-day class, participants were led in meditation, taught didactic information, practiced techniques, and demonstrated the techniques with each other. The didactic portion of the HT class covers essential topics, including the definition of HT, evidence-based benefits, and its theoretical foundation. Additional subjects include the physiologic responses to energy healing, a foundational understanding of the human energy system, and methods for assessing it. The course also emphasizes practitioner self-care and teaches 12 energetic techniques, including the SCC.^14^

The SCC technique was taught and practiced as a meditation on the second day of class. The SCC technique is a full-body self-care method used to clear, connect, and balance the energy systems, promoting relaxation, calmness, and mental clarity.^13^,^14^ During the SCC, the individual places their hands in a sequential pattern over the major and minor energy centers on the body. The sequential hand placements move up the body, starting at the feet and legs, through the torso, including the arms, and ending at the top of the head. SCC can be used at any time, in any environment, and can be variable in length.^13^,^14^

**Table:** Baseline characteristics

	N	%
**Sex**		
Female	33	97.06
Male	1	2.94
**Age**		
25-34 years old	11	32.35
35-44 years old	14	41.18
45-54 years old	6	17.65
55-64 years old	3	8.82
**Degree∗**		
Associates	2	6.06
BSN	25	75.76
MSN	6	18.18
**Area of nursing practice**		
ICU	1	2.94
ED	3	8.82
Behavioral health	2	5.88
Medical floor	11	32.35
Surgical prep/PACU	1	2.94
Other (case manager, diabetes educator, nurse manager, nursing professional development specialist, nursing supervisor, wound ostomy continence nurse)	16	47.06
**Ethnic background**		
Asian	3	8.82
Black or African American	4	11.76
Prefer not to answer	1	2.94
White	26	76.47
**Hourly work status**		
Full-time	29	85.29
Part-time	3	8.82
Per diem	1	2.94
WIP (weekend incentive program)	1	2.94
**Typical shift worked**		
Days	28	82.35
Evenings	2	5.88
Nights	4	11.76

∗ One participant did not complete this question.

### Data collection

Upon opting into the study, participants were invited to complete a secure electronic survey before class. The survey included consent, the Perceived Stress Scale (PSS), and a demographic data questionnaire. After completing the class, participants were instructed to perform the SCC for 21 days as a self-care practice. Twenty-one days after the class concluded, participants received the electronic post-survey, which included Cohen's PSS and three open-ended qualitative questions.

Cohen's PSS, a validated 10-item questionnaire, measures how different situations affect feelings and perceived stress (see *Perceived Stress Scale*).^26^ Individual scores on the PSS can range from 0 to 40, with higher scores indicating higher perceived stress. Scores ranging from 0 to 13 would be considered low stress. Scores ranging from 14 to 26 would be considered moderate stress. Scores ranging from 27 to 40 would be considered high perceived stress.^26^ The post-survey included three open-ended questions: “How has taking the Healing Touch class assisted you in your daily life?”; “After integrating the self-chakra connection into your daily practice, how have you changed?”; and “After taking the Healing Touch classes and implementing the self-chakra connection into my daily self-care practice, how have I found real enjoyment in my work on my nursing unit? Here are examples of enjoyment that I have experienced in my nursing practice.”

The investigators consulted with the organization's Institutional Review Board (IRB) and were advised to seek an expedited review. The IRB assessed the study and granted it exemption from oversight under exempt category 45 CFR 46.104(d)(2)(iii), with a Limited IRB Review under 45 CFR 46.111(a)(7). Provisions were made to protect the privacy of subjects and the confidentiality of data. Participants anonymously filled out the demographic and PSS scale via a Microsoft Forms link. The confidentiality of subjects and data were preserved by storing the completed electronic forms on a password-protected computer. No personal information was collected, and the data were not examined until after the study concluded. Throughout the study, policies and procedures were followed to obtain consent and ensure ethical conduct.

### Analysis

Descriptive and correlational statistics were used to evaluate the impact of the HT intervention in the study. Participant demographics were summarized using frequency (N, %) for categorical variables (sex, age, nursing degree) (see *Baseline characteristics*). Differences between pre- and post-PSS scores were summarized using sample size, mean, and median scores (see *PSS scores*). One-tailed F-test was used to determine a significant mean effect for the treatment of the pre- and post-groups, and that they were equal to the same value, with a significance set at *P* <.05 (see *F-Test variance*).^27^,^28^ Analyses were conducted using SAS version 9.4 (SAS Institute Inc.)^29^ and Microsoft^®^ Excel^®^ for Microsoft 365 MSO (Version 2302 Build 16.0.16130.20806) 32-bit.^30^

## Results

Fifty-eight nurses participated in the HT classes from October 2022 to January 2023. Thirty-four nurses completed the presurvey (59%) of which 22 also completed the postsurvey (38%).

Most participants were female (33, 97%), with one male participant (3%). Participants ranged in age from 25 to 64 years old, with 41 reporting age in the 35 to 44 range. Educational preparation varied, with most participants reporting a bachelor's degree as the highest level of education (76%).

**Table:** PSS scores

	N	Mean	Median	SD	Range
Baseline	34	18.1	19.5	6	10
Post	22	14.3	14.5	5.3	9
Change from baseline		-3.8	-5		-1

Footnote: Participants were asked to place their unique code for identification in the pre- and post-survey. Several post-survey participants were not able to remember their unique code, so matched t-tests were not completed.

The baseline mean PSS score across the sample was 18.1 (median of 19.5) and a standard deviation of 6. Of the 22 nurses completing the follow-up at both time points, there was a 3.8-point decrease in the mean (median decrease of 5 points) PSS score from baseline to follow-up. The nurses' baseline and follow-up PSS scores were 18.1 and 14.3, respectively.

Additionally, the PSS results demonstrated that the nurses experienced increased confidence in their ability to handle personal problems, reported that things were going their way, felt the ability to control irritations, and experienced being on top of things. Nurses stated that they utilized the SCC technique to start their day feeling grounded, centered, connected, and energized, while others used it before bed to promote relaxation and sleep.

**Table:** F-test variance

	Pre-survey	Post-survey
Mean	18.14706	14.31818182
Variance	36.06863	27.94155844
Observations	34	22
df	33	21
F	1.29086	
P(F<=f) one-tail	0.272767	
F Critical one-tail	1.993862	

Footnote: Participants were asked to place their unique code for identification in the pre- and post-survey. Several post-survey participants were not able to remember their unique code. One-tailed F-test was used to demonstrate the mean effect for the pre- and post-survey groups.

### Post-open-ended questions

Responses to the open-ended questions indicated that the nurses felt that HT helped them relax and take time out (see *Selected participant quotes*). Participants reported that HT facilitated their return to caregiving responsibilities, helping them feel ready to care for patients more effectively. Additionally, participants reported that HT helped them understand that it only takes a few minutes to ground and center in one's body to become focused and calm. They also reported that they later felt more prepared for their shift, ready to start fresh.

## Discussion

Data analysis of the PSS demonstrated trends toward decreased perceived stress, upset feelings, anxiety, and nervousness among participants. Nurses noted improved feelings of being able to handle life's challenges.

This study's findings suggest that HT classes and self-care techniques are associated with improved self-reported perceived stress scores as part of a self-care routine. These results are consistent with other biofield therapy studies on other populations, suggesting lower stress levels with learning, and practicing HT.^9^

However, statistical significance was not achieved. Due to the pilot nature of the study, one potential reason for lack of significance is low post-survey completion of 22 responses. Possible reasons for attrition are based on speculation: participants did not practice SCC during the 21-day period, lack of interest, no effects noted by participants, or misplacing the email with the follow-up questionnaire link. For future studies, data collection would likely be supported by education on the importance of returning the survey completion regardless of the consistency of practice or the noted impact of the SCC practice.

For example, these results are comparable to those of Cuneo et al.^31^ about the effect of Reiki, a complementary energy practice that originated in Japan, on work-related stress of RNs. The investigators studied the perceived stress levels of nurses before and after learning and using self-Reiki techniques. They noted a mean 5.9-point decrease and medium decrease of 7 points in the PSS scores from baseline to follow-up.

For hospitals or health systems exploring how best to invest in their nurses' well-being, developing HT programs and providing training may be one potential complementary approach to improving outcomes. However, additional large-scale studies are needed to fully explore the impact of these practices. Additionally, skepticism exists within the medical community regarding the effectiveness of HT, due to the lack of standardization in study methodologies and the subjective nature of some reported outcomes. Addressing these gaps through controlled trials and large-scale studies will be essential to potentially gain broader acceptance of HT as a complementary healthcare modality.

## Limitations

The study had several limitations, including the small sample size and post-intervention survey respondents. The participants were asked to place their unique identification code for the pre- and post-survey. However, several post-survey participants did not remember their unique codes, so a matched t-test was not conducted. A one-tailed F test was done due to the small size.

No sample size calculation was completed before the study, and as such, it was not adequately powered to detect small changes in self-reported outcomes. The lack of randomization and a control group is a limitation; therefore, observed effects cannot be attributed solely to HT training and practice alone. The study utilized a convenience sample of self-selected participants which may not represent the general nursing population. Lastly, the study was conducted at a single hospital site and may not be generalizable to other institutions or hospitals.

## Recommendations for future studies

Further research is warranted. Performing a larger randomized controlled study comparing the effects of the practice of the SCC on work-related stress would provide actionable information. Other possible areas for future research include examining the effects of HT education and training on nurse recruitment and retention and measuring patient satisfaction in patient-care units where nurses practice SCC and provide HT for their patients. It may be worthwhile in future studies to compare HT training with another type of stress management training, such as mindfulness-based stress reduction.

## Conclusion

Nurses may experience mental, emotional, or physical stress secondary to work demands, high-stress environments, or traumatic experiences. Nurses need to have a positive sense of well-being and easy-to-use self-care methods to ensure the highest quality of care to patients and themselves.

HT is a holistic energy healing therapy in which the practitioner consciously uses their hands in a heart-centered and intentional way to enhance, support, and facilitate physical, mental, emotional, and spiritual health and self-healing. HT education and practice are promoted by the American Holistic Nurses Association, the HTP, the HT Professional Association, certain collegiate nursing school curriculums, and hospitals that utilize HT as a holistic nursing practice to care for staff and patients. Another strategy is SCC, which is a self-care technique used to facilitate the balancing of the human energy system to promote relaxation.

This pilot study found that nurses who received HT training and practiced the SCC as part of their self-care reported decreased perceived stress levels. The results of this study support HT training and the use of the techniques as one approach for stress reduction among nurses.

## Selected participant quotes

- “The self-chakra connection technique is an easy self-care activity that I do each night to help me fall asleep quickly and easily.”
- “I use the self-chakra connection technique in the morning. It helps me to start the day feeling calm, centered, and grounded.”
- “I can perform Healing Touch on the patients, and they feel like I am doing something special for them that they didn't expect and that is allowing for a more special connection between myself and the patients.”
- “It has made me look at things in a different light. Try and take the good rather than the bad. When everything seems to be going wrong, I just take the time out. You always think you need all this time to relax or calm yourself, but healing touch helped me understand that it only take a few minutes.”
- “I am able to offer alternatives for patients who don't want medication to relax.”
- “I have felt more prepared for my shift; ready to start fresh instead of bringing my bad energy or feelings I may have at home to work; patients are more open and willing to participate in nursing activities after interacting with me.”
